# Developing a delivery science for artificial intelligence in healthcare

**DOI:** 10.1038/s41746-020-00318-y

**Published:** 2020-08-21

**Authors:** Ron C. Li, Steven M. Asch, Nigam H. Shah

**Affiliations:** 1 Division of Hospital Medicine, Department of Medicine, Stanford University School of Medicine, Stanford, CA USA; 2 Center for Biomedical Informatics Research, Department of Medicine, Stanford University School of Medicine, Stanford, CA USA; 3 Division of Primary Care and Population Health, Department of Medicine, Stanford University School of Medicine, Stanford, CA USA; 4 Center for Innovation to Implementation, Department of Veterans Affairs, Palo Alto, CA USA

**Keywords:** Translational research, Health services

## Abstract

Artificial Intelligence (AI) has generated a large amount of excitement in healthcare, mostly driven by the emergence of increasingly accurate machine learning models. However, the promise of AI delivering scalable and sustained value for patient care in the real world setting has yet to be realized. In order to safely and effectively bring AI into use in healthcare, there needs to be a concerted effort around not just the creation, but also the delivery of AI. This AI “delivery science” will require a broader set of tools, such as design thinking, process improvement, and implementation science, as well as a broader definition of what AI will look like in practice, which includes not just machine learning models and their predictions, but also the new systems for care delivery that they enable. The careful design, implementation, and evaluation of these AI enabled systems will be important in the effort to understand how AI can improve healthcare.

Artificial intelligence (AI) has generated much excitement, but relatively little impact in how healthcare is delivered. While progress has accelerated in using machine learning (ML) to develop prediction and classification models that make up the bulk of current AI methods^1^, efforts to use these models in the real world setting have not taken off at nearly the same pace^2^ and typically remain within the realm of “innovation” outside of the core processes that drive care delivery^3^. To address how AI can be leveraged at scale, we need to both broaden and deepen our thinking around how AI fits into the complexities of healthcare delivery. As the data and computer sciences for developing AI based solutions have matured, we now need a *delivery* science to bring those solutions into use in healthcare.

Current efforts to use AI in healthcare often begin with “I have a ML model that can accurately predict or classify X”, but then get stuck at “how do I use it and for whom?”^4^ As a result, libraries of ML models remain on the shelf without finding appropriate use cases, or models are implemented but deemed to not be as valuable as initially imagined^5^. A recently published ML model that predicts acute kidney injury with high accuracy^6^ was assumed by the authors to provide valuable information to clinicians, but when implemented in a real clinical environment, did not significantly improve patient care and in fact resulted in additional work for the physicians that was of unclear value^7^. This example highlights the importance of understanding the complexities of care delivery associated with the clinical use case *before* building the ML model; just focusing on the capability to accurately perform a prediction task is not sufficient for improving care. This conundrum is not unique to AI; it frequently affects innovation pipelines for other biomedical technologies. For example, the lengthy and rigorous process required in drug development from preclinical experiments to observed health benefits in the real world illustrate the amount of work needed to translate scientific advances into useful therapies that actually improve care^8^. Much of the in silico work around training and validating ML models can be compared to the preclinical testing of active ingredients in pharmaceutical research. Just as the active ingredient alone is not sufficient for creating a drug that works in humans, much less a clinical intervention that improves outcomes for a patient population, a ML model alone is unlikely to make significant improvements in healthcare outcomes.

It is time to move from model development in silico to design, implementation, and evaluation of AI enabled solutions in vivo where healthcare delivery happens. We propose a delivery science for AI in healthcare that rests on the following principles: (1) much of healthcare is delivered in complex adaptive systems^9^, so AI must accommodate this complexity, (2) AI should be viewed as not the end product, but rather an *enabling* component of broader solutions, and (3) solutions enabled by AI are often complex systems of people, processes, and technologies. We need to take a more holistic view of what AI *enabled* solutions would look like beyond just a set of ML models. Rather, the human and technical components of the end product, such as the workflows, teams, and digital tools made possible by tasks that a ML model can perform, should be designed and implemented together as a system. The effects—beneficial or harmful—of AI enabled solutions on healthcare should also be evaluated at the system level as emergent properties that may be greater than the sum of its individual components. Identifying these emergent properties and characterizing their impact will require the system to be designed and implemented in its entirety in the healthcare environment where it is meant to operate. The task of implementing AI in healthcare, therefore, should not be about deploying a ML model; rather, it should be about how to design the best possible care delivery system for a given problem, using the ML model as a component in that delivery system.

Our initial experiences with the design, implementation, and evaluation of an AI enabled solution at an academic medical center has revealed the importance of marrying data science with disciplines, such as process improvement, design thinking, and implementation science (Fig. 1). We had previously developed an all-cause mortality prediction model to act as a proxy for who may benefit from palliative care services such as advance care planning^10^. Rather than jumping to a solution of simply showing the model output to physicians, we first leveraged methods from process improvement to derive the sources of process inefficiencies and breakdowns^11^, and design thinking to observe how these processes affected the thoughts, feelings, and experiences of frontline stakeholders^12^. These steps allowed us to first understand the complex system in which advance care planning is currently delivered before designing a solution enabled by our ML model that could improve on that delivery system.

**Fig. 1:**
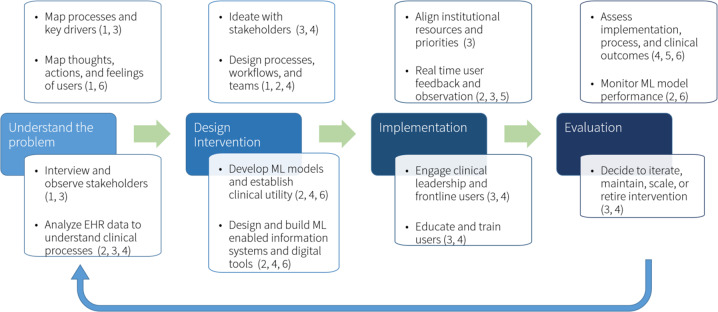
Multidisciplinary process for creating, implementing, and evaluating an AI enabled system for healthcare. Methods from process improvement, design thinking, data science, information technology, and implementation science are combined into an iterative participatory process to build an AI enabled system for improving advance care planning. The expertize used across the different disciplines are as follows: (1) user experience design, (2) data science, (3) healthcare operations, (4) clinical informatics, (5) evaluation, and (6) ethical integrity assessment.

We made a key decision upfront to engage a multidisciplinary group of stakeholders, including frontline nurses, physicians, social workers, and occupational therapists—all who participate in the care of patients with serious illnesses who may benefit from advance care planning—from the beginning of the design process without any preconceived notions of how the ML model would be used. Interviews and process analyses of the current state quickly revealed key barriers to advance care planning that would unlikely be solved by simply showing a model’s output to any one group of clinicians. For example, clinical and logistical considerations around the appropriate timing of advance care planning, what should be discussed, and how should these discussions fit into the broader context of the hospitalization require coordinated, multidisciplinary efforts. Similarly, design thinking tools such as empathy mapping^13^ helped us more deeply understand how underlying feelings around role clarity and power structures between physician and non-physician members of the care team affected advance care planning workflow. These insights led us to identify key design goals that otherwise would not have surfaced, such as the need to empower non-physician care team members to identify candidate patients and lead the coordination of advance care planning—a task that was enabled by making transparent to the entire care team the list of candidate patients generated by the mortality prediction model each day and creating a workflow for the physician and non-physician team members to discuss these patients with each other about advance care planning needs. This objective identification of candidate patients by the prediction model allowed for the democratization of responsibility for deciding who needs advance care planning to the non-physician providers such as nurses, social workers, and occupational therapists—all who spend a lot of time with patients and are trained to engage in this topic. The design process also includes analyses to verify that our model’s execution and runtime characteristics (such when in the day are predictions available) fit the logistical needs of these new workflows^14^. This deeper understanding of current state gaps and improvement opportunities allowed us to build a system of workflows, teams, and digital tools *enabled* by the mortality prediction model to drive change in the complex environment of healthcare delivery. Other AI efforts that address the broader sociotechnical components of healthcare beyond just the ML model have offered similar lessons. For example, recent work around using AI to improve the treatment of sepsis committed months to assessing clinical processes and user experiences prior to even training a ML model, which yielded important insights for implementation, such as the need to focus on not just sepsis detection, but a method for standardizing follow up care^15^.

To be most useful, evaluations of AI enabled solutions should not simply ask whether it achieved the desired improvement in clinical process or outcome (e.g., did the frequency or quality of advance care planning improve), but also how well or poorly was the solution implemented. Implementation science and systems engineering tell us that we can use rigorous scientific methods for both effectiveness and implementation questions. Such hybrid evaluations can assess the mechanism(s) by which AI enables the changes that lead to the desired clinical outcome (how did the mortality prediction tasks performed by the ML model mediate the improvement in advance care planning) and the properties of the overall AI enabled systems (what are the structures, patterns, and processes of the workflows, teams, and technologies that make up the new AI enabled system for delivering advance care planning). Frameworks such as RE-AIM^13^ (reach, effectiveness, adoption, implementation, and maintenance) can help identify the dimensions by which to assess implementation and subsequent dissemination efforts, and models for sociotechnical systems such as SEIPS^14^ (Systems Engineering Initiative for Patient Safety) can help assess the complex interactions between people and technologies in a work system.

Naturally, questions about who is responsible for implementing such delivery systems, and quality control of the ML workflows will arise. Aside from existing processes in healthcare systems to design standard operating procedures, additional attention will be needed to implement quality controls on the models itself. Specifically, to monitor a model’s calibration over time, it will be important to watch population drifts and ensure timely retraining so that the model’s performance remains with in the execution and runtime characteristics required by the AI enabled system^16,17^. Just as with clinical laboratory instruments, ML models in healthcare will need to be regularly re-calibrated and tuned. The characteristics of the ML models will also need to be appropriately communicated to clinical users^18^. Fortunately, there is deep experience in the technology sector to draw upon^19,20^. In-house informatics teams within health systems with expertize in data science, information technology, and clinical operations may be required to own this work. While the nature of these teams may vary across organizations, what is certain is that such a team will need to exist to ensure that AI will be used responsibly and deliver sustained value.

It is time to move AI research out from in silico model development into real world design, implementation, and evaluation for improving healthcare delivery. We will likely see that ML models will be necessary, but not sufficient components of broader AI enabled solutions. The delivery science of AI will need to address how such systems are designed, implemented, and evaluated, and how their emergent properties can be captured and utilized to transform healthcare.
